# Hepatic breast cancer dissemination after an iatrogenic hepatic laceration during talc pleurodesis: a case report

**DOI:** 10.1186/1755-7682-3-6

**Published:** 2010-05-06

**Authors:** Joaquim Bosch-Barrera, Jaime Espinós

**Affiliations:** 1 Department of Oncology, Clínica Universidad de Navarra, Universidad de Navarra, Calle Pío XII 36, 31008, Pamplona, Spain

## Abstract

**Background:**

Talc pleurodesis is an effective treatment for malignant pleural effusion. We present a case of an asymptomatic hepatic laceration that occurred during pleurodesis in a breast cancer patient and led to hepatic tumor dissemination.

**Discussion:**

Pleurodesis is a relatively safe procedure, although previous studies have described malignant invasion of scar tissue.

**Conclusion:**

To our knowledge, this is the first case report of tumor spread due to a liver puncture during talc pleurodesis in a breast cancer patient.

## Background

Malignant pleural effusion (MPE) is common in patients with advanced cancer [1]. The primary neoplasms that most frequently cause MPE are lung cancer in men and breast cancer in women. Talc pleurodesis is a widely used technique for the treatment of MPE [2]. We present a case report of an asymptomatic complication during talc pleurodesis that caused a hepatic dissemination of a breast tumor that was eventually fatal.

## Case presentation

A 59-year-old woman was admitted to her reference hospital for dyspnea. Chest radiography showed a severe right pleural effusion. Her medical history included breast cancer (pT2pN1M0) diagnosed when she was 52, which was treated with surgery, adjuvant chemotherapy, adjuvant radiotherapy and hormonotherapy (3 years of tamoxifen and 2 years of letrozole). The patient did not have other diseases or congenital malformations and had not required previous abdominal or thoracic surgeries. The last normal follow-up was 2 months prior to her admission for the dyspnea. A thoracic tube was inserted, and 1,000 cc of pleural fluid was drained. After cytology revealed the presence of malignant breast cells, right-sided talc pleurodesis was performed. The patient had a good postoperative evolution, without fever or pain, and was discharged four days after the talc pleurodesis.

**Figure 1:**
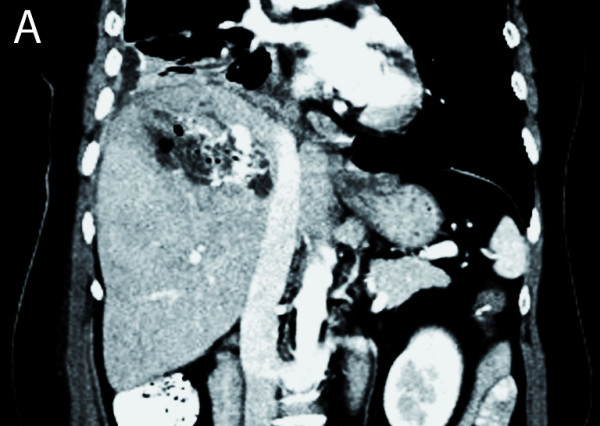
**A heterogeneous hepatic lesion with air and dense content (talc) was observed after talc injection into the liver.No hepatic metastasis was observed at this time.**

**Figure 2:**
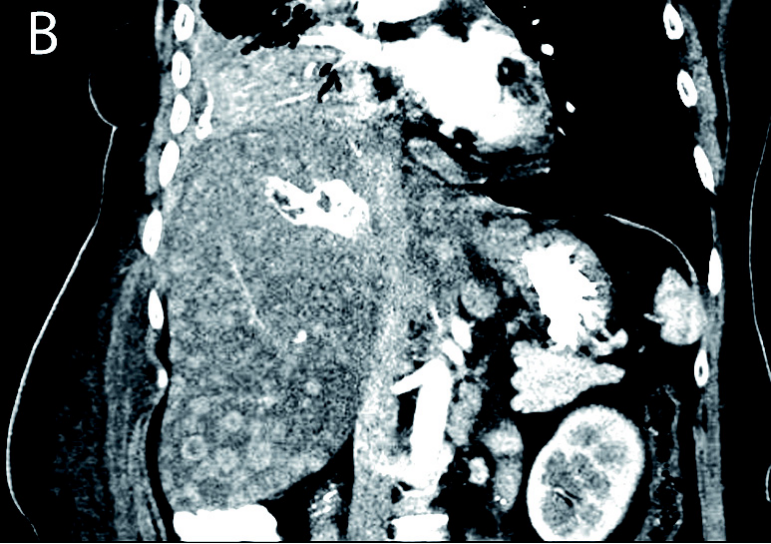
**A dense region corresponding to the site of talc injection was observed one month later. Multiple liver metastases were also observed**.

The patient came to our center for oncological treatment. A staging CT scan showed a heterogeneous hepatic lesion (9 × 6.5 cm) with air inside (Fig. 1). In addition, bone and pleural affectation from the patient's metastatic breast cancer were observed. The patient was asymptomatic, without fever, abdominal pain or abnormal laboratory tests. A hepatic abscess was ruled out because of her clinical history, and a hepatic laceration made during the talc-injection procedure was suspected. Hormonal treatment (exemestane) was started. A new CT scan was performed one month later. A diffuse malignant hepatic infiltration was observed (Fig. 2) without any tumor progression at distal sites. Laboratory analysis yielded the following values: bilirubin 1.66 mg/dL; GOT 146 UI/L; GPT 112 UI/L; LDH 2,934 UI/L; GGT 1,405 UI/L; and alkaline phosphatase 1,383 UI/L. An initial cycle of 50 mg/m^2 ^doxorubicin hydrochloride and 500 mg/m^2 ^cyclophosphamide was administered. The patient died four weeks later as a result of hepatic failure.

## Discussion

Talc pleurodesis is recognized as the procedure of choice for the treatment of MPE [2], and a meta-analysis has confirmed the superiority of talc as a sclerosing agent [3]. However, some studies have generated concerns about the safety of the procedure [4-6]. The adverse effects reported include mild fever, chest pain and, more rarely, acute respiratory distress syndrome (ARDS), empyema, wound infection and malignant invasion of scar tissue [7,8]. To our knowledge, this is the first case reporting a hepatic laceration due to talc pleurodesis followed by hepatic tumor spread.

A CT scan performed before of the pleurodesis did not show any hepatic lesion and liver tests were normal in that moment. In this patient, the initial diagnostic suspicion of a hepatic abscess was supported by the CT image, but normal blood tests and the absence of clinical symptoms ruled it out after 24 hours of observation. Consequently hormonal treatment was preferred over chemotherapy to avoid immunosuppression, which could have complicated the treatment of a hepatic infection.

We presumed that the advanced malignant hepatic progression was secondary to breast tumor cell dissemination due to the introduction of malignant pleural effusate into the liver. This condition could also have been the result of systemic dissemination, but no other locations exhibited disease progression and the hepatic dissemination was surprisingly rapid. Moreover, malignant invasion of scar tissue after talc pleurodesis has been described previously [5]. Another possible explanation for the presence of tumor cells in the abdomen could be a direct communication between the abdominal cavity and thorax due to a diaphragmatic defect, but our patient did not have previous diaphragmatic defects. In 1998, Kirschner described the porous diaphragm syndrome (PDS) [9]. The PDS is connected to the pass from the peritoneal cavity to the pleural space. We consider that the absence of ascites during the course of the disease and the presence of intrahepatic metastases without implants in the liver capsule excludes this possibility in our case.

## Conclusions

To our knowledge, this is the first case report of tumor spread due to a liver puncture during talc pleurodesis in a breast cancer patient. This complication must be taken into account when pleurodesis is performed.

## Abbreviations

MPE: malignant pleural effusion; CT: Computed Tomography; GOT: glutamic-oxaloacetic transaminase; GPT: Glutamic-pyruvic transaminase; LDH: Lactate dehydrogenase; GGT: Gamma glutamyl transpeptidase.

## Consent

Written informed consent was obtained from the patient's relatives for publication of this case report and any accompanying images. A copy of the written consent is available for review by the Editor-in-Chief of this journal.

## Competing interests

The authors declare that they have no competing interests.

## Authors' contributions

JB-B developed the concept and wrote the draft. JB-B and JE contributed to the final manuscript version and have given final approval of this version.
